# Irritation of the Fauces with Pain in the Shoulders and Thorase, Caused from Periostial Inflamation of the Teeth

**Published:** 1865-07

**Authors:** Julius Chesebrough


### Correspondence.

---

### IRRITATION OF THE FAUCES, WITH PAIN IN THE SHOULDERS AND THORAX, CAUSE FROM PERIOSTIAL INFLAMATION OF THE TEETH.

Mr. Editor.—A singular case was presented to me for advice in relation to an extreme irritation of the fauces, and a soreness of the teeth. Upon questioning the party, I found that about the first of February, she wet her feet and the result caused a slight soreness of her teeth. This soon passed away, and the day following she complained of soreness of the fauces.

She was treated by her physician (?) for a "scrofulous irritation." Thus she has been until she presented herself to me, May 12th. On examination I found the fauces turgid and emitting sanies and pus. The secretions were very acrid and irritating and the mucous membrane was mostly abbraded. She experienced great soreness about the shoulders, and when the pain left that part, she had an oppressive sensation in the thorax. The teeth were very loose, and the case resolved itself at once.

These organs were the seat of the whole trouble. Though sound and without blemish, I could but rely upon their being the domant actor upon the other parts. I at once ordered,

Potass Iodii grs. xl.

Syrupus Simp. ℥ iii.

Ess. Gaultheria ℥ ss.

Tablespoonful every two hours.

To correct the discharge from the mucous surface, and to deprive it of its virulent property, ordered,

Potass Chloras ℥ i.

Aqua ℥ iv.

to use as a gargle every half hour. The result in so short a time was highly satisfactory, for in 24 hours the discharge had ceased and the teeth had occupied their former position in the alveolar process, and in twelve hours more no vestage of the trouble remained, but the condition of the mucous membrane, which is at this time entirely healed.

This case has brought to mind similar cases, where the physician in charge has erred in his treatment from ignorance of the relation of the teeth to other parts of the body. This case was none other than a periostial inflamation which had extended itself until it comprised within its bounds the mucous membrane of the month and fauces, and he (graduate of medicine) called it "scrofulous irritation." Shade of Hippocrates from your dwelling place, come to earth, once more and give your descendants common sense, that they may learn and discover that the error of their lives, is their imperfect knowledge of the formation, structure, function and sympathy of the teeth.

JULIUS CHESEBROUGH, D. D. S.

May 17th, 1865.

